# Introduced species and extreme weather as key drivers of reproductive output in three sympatric albatrosses

**DOI:** 10.1038/s41598-020-64662-5

**Published:** 2020-05-18

**Authors:** Jaimie B. Cleeland, Deborah Pardo, Ben Raymond, Aleks Terauds, Rachael Alderman, Clive R. McMahon, Richard A. Phillips, Mary-Anne Lea, Mark A. Hindell

**Affiliations:** 1 Institute for Marine and Antarctic Studies, University of Tasmania, 20 Castray Esplanade, Battery Point, Tasmania 7004 Australia; 2 Australian Antarctic Division, Department of Agriculture, Water and the Environment, 203 Channel Highway, Kingston, Tasmania 7050 Australia; 3 British Antarctic Survey, Natural Environment Research Council, High Cross, Madingley Road, Cambridge, CB3 0ET UK; 4 Department of Primary Industries, Parks, Water and Environment, Hobart, 7000 Tasmania Australia; 5 Sydney Institute of Marine Science, 19 Chowder Bay Road, Mosman, New South Wales 2088 Australia

**Keywords:** Population dynamics, Conservation biology, Invasive species

## Abstract

Invasive species present a major conservation threat globally and nowhere are their affects more pronounced than in island ecosystems. Determining how native island populations respond demographically to invasive species can provide information to mitigate the negative effects of invasive species. Using 20 years of mark-recapture data from three sympatric species of albatrosses (black-browed *Thalassarche melanophris*, grey-headed *T. chrysostoma*, and light-mantled albatrosses *Phoebetria palpebrata*), we quantified the influence of invasive European rabbits *Oryctolagus cuniculus* and extreme weather patterns on breeding probability and success. Temporal variability in rabbit density explained 33–76% of the variability in breeding probability for all three species, with severe decreases in breeding probability observed after a lag period following highest rabbit numbers. For black-browed albatrosses, the combination of extreme rainfall and high rabbit density explained 33% of total trait variability and dramatically reduced breeding success. We showed that invasive rabbits and extreme weather events reduce reproductive output in albatrosses and that eliminating rabbits had a positive effect on albatross reproduction. This illustrates how active animal management at a local breeding site can result in positive population outcomes even for wide ranging animals like albatrosses where influencing vital rates during their at-sea migrations is more challenging.

## Introduction

Invasive species are a major threat to global biodiversity, with the capacity to cause rapid ecosystem change^1,2^. Island ecosystems, which generally show high levels of endemism, are particularly vulnerable to the direct and indirect stressors of invasive species, including competition, predation and habitat degradation^3^. The influence of invasive species on island ecosystems already experiencing pressure from global climate change may result in trophic imbalances amongst native communities, and reduced species resilience in the face of multiple threats^4^. Further, given the frequency and severity of extreme weather events, such as heat waves and precipitation, are predicted to increase as a result of global climate change^5^, their effects on island ecosystem structure and function are likely to be exacerbated^6^. In severe cases, the combination of invasive species and extreme weather events could have particularly serious demographic consequences for threatened native species.

Amongst invasive species, introduced mammals are particularly problematic^7^. The defining characteristics of the most successful mammalian invaders are high adaptability and rapid reproduction, both of which increase the likelihood of population establishment and the rate of dispersal^8^. The European rabbit *Oryctolagus cuniculus* epitomises these life history traits, contributing to its historical dominance as a successful invasive species worldwide^9^. Acting as an ecosystem engineer, rabbits can reduce landscape heterogeneity through over-grazing that can lead to catastrophic habitat degradation^10^. Rabbit populations typically grow quickly before decreasing rapidly once local carrying capacity is reached in a boom and bust scenario^11,12^. The transitional period between the boom and the bust can swiftly transform a landscape through high vegetation suppression and increased erosion rates and consequently, drastically degrade native species communities^13,14^.

Invasive rabbits on islands often co-exist with native seabird colonies^15,16^, with vegetation providing fodder for rabbits and nesting habitat for seabirds. There are profound demographic responses of burrowing seabirds to high rabbit density, including reduced reproductive output through increased competition for burrow-nesting habitat^17,18^. However, for surface-nesting seabirds such as albatrosses, the consequences of high rabbit density and associated habitat change remain unquantified. As rabbit population densities grow, elevated grazing pressure can lead to a reduction in vegetation cover and plant diversity^19^, with the potential to deplete available nesting material, increase predation risk, or increase variability in nest microclimate. For surface-nesting seabirds greater exposure to weather conditions may increase the energetic costs of incubation or brooding, and for unattended chicks increase thermoregulatory costs^20,21^. With greater exposure, the risk of predation is also likely to increase, as adults, eggs and chicks become more visible^22^. At high rabbit densities, increased soil erosion rates driven by severe vegetation suppression may also influence nest site selection, as some areas become less suitable^13^.

Typically, in long-lived species such as albatrosses, that inhabit variable environments, fluctuations in breeding probability and success often have limited influence on regulating population growth rates. However, for albatrosses which have low immigration rates and in most cases severely diminished populations due to widescale declines in adult survival driven by interactions with commercial fishing operations^23^, reproductive rates can have greater influence on population growth rate and should be assessed.

Subantarctic Macquarie Island has experienced drastic change over the last ~140 years due to the establishment and proliferation of invasive species. First introduced by pre-industrial commercial sealers in the late 19^th^ century; cat *Felis catus*, weka *Gallirallus australis scotti*, rabbit, rat *Rattus rattus* and mouse *Mus musculus* populations have fluctuated due to variability in resource availability and more recently through management interventions^24,25^. With coordinated releases of the rabbit control virus Myxoma, rabbit numbers were reduced from around 300,000 in 1977 to 30,000 or less by the late 1980s, remaining low until the late 1990s^12^. A subsequent rapid rise in rabbit numbers between 2001 and 2005 has been attributed to multiple factors including the eradication of feral cats, reduced distribution and effectiveness of the Myxoma virus, warmer climatic conditions and increased availability of food resources as a result of twenty years of vegetation recovery following a myxo-induced reduction of the rabbit population^2,26,27^. Before they were eradicated in 2000, feral cats preyed heavily on rabbits and burrowing petrels, using both as a main prey source^17,28^. However, there are no reliable indications that cats regularly preyed upon nesting albatrosses, possibly due a surplus of preferred prey and an effective defence mechanism: the regurgitation of stomach oil^28^. Furthermore, intensive control of cats began in 1996, two years after the initiation of this study, with cage trapping being the most effective method for considerably reducing the population^29^. While rats have contributed to breeding failure of several burrowing petrel species on Macquarie Island, there is no clear evidence that they depredate albatross eggs or chicks in active nests^30,31^. In 2014, one of the largest and most ambitious invasive species eradications to date was deemed successful in removing rabbits, rats and mice from Macquarie Island^32^. While present on the island, rabbits caused widespread and severe vegetation suppression, enhanced slope erosion and more frequent landslide events (see Supplementary Figs. S1 and S2)^13,14^. Spatial and temporal patterns in rabbit density and subsequent habitat degradation were not uniform across the island. Patterns in rabbit density show numbers reaching a peak in smaller, localised areas before declining, representing the carrying capacity of the area and depletion of available food^12^. The peak in rabbit density in the north of Macquarie Island (2005) occurred approximately four and a half years prior to the peak in the middle and south of the island (2009)^12^. Furthermore, rabbit impacts have coincided with changes in climate: increases in surface air temperatures by 0.62 °C (1948–2007), total annual precipitation by 35%, and higher wind speeds^33–35^. Rising annual rainfall has the capacity to increase the frequency and extent of landslips, and the rate of erosion on Macquarie Island’s coastal slopes, which has been made worse in recent years by the widespread loss of vegetation from rabbit grazing^13^.

**Figure 1:**
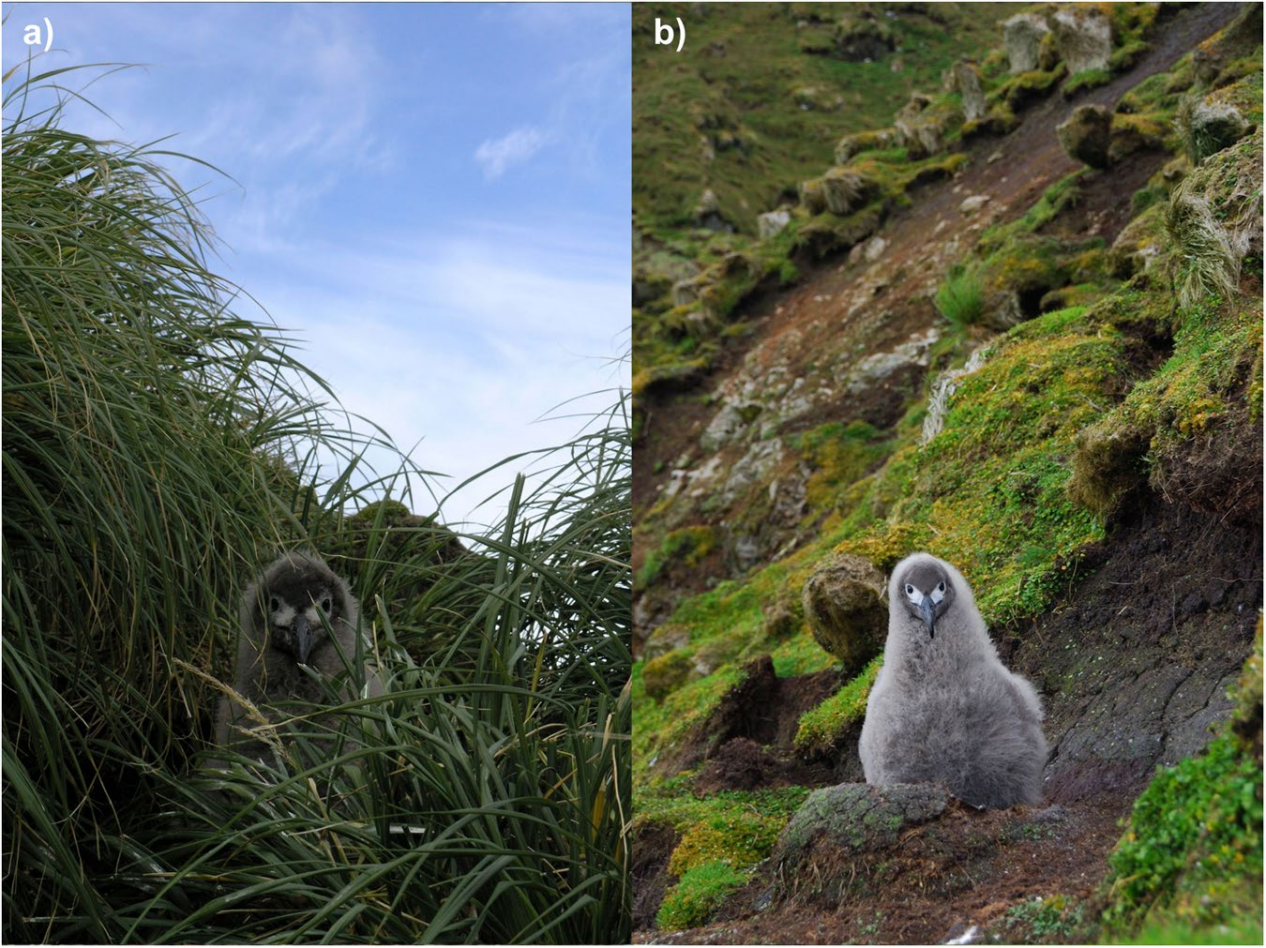
Rabbit free and rabbit grazed albatross habitat. Photos illustrating nest exposure and microclimate differences of (**a**) a pre-fledging light-mantled albatross chick nesting in a region of Macquarie Island protected by a rabbit-proof fence and regular control (date taken: 2006-04-11, source: R. Trebilco) compared to (**b**) a well-grown chick in a heavily rabbit-grazed region in the south-east of Macquarie Island (date taken: 2007-02-24, source: R. Trebilco).

Four albatross species breed on Macquarie Island, of which three; black-browed *Thalassarche melanophris*, grey-headed *T. chrysostoma* and light-mantled albatrosses *Phoebetria palpebratra* nest on high, exposed escarpments. These steep nest sites have experienced major degradation driven by over-grazing and landslips from resulting soil instability, geological activity and heavy rainfall events (Fig. 1). Quantifying the progressive influence of invasive species on the demographic rates of native island species is often hampered by the lack of available data on invasive species populations. Additionally, understanding the medium to long-term demographic effect of extreme weather conditions requires long-term biological datasets. Together, these challenges mean that few studies have addressed the synergistic effects of both invasive species and extreme weather.

We quantified the influences of rabbit density and heavy rainfall events on the breeding probability and breeding success of these three albatross species. Understanding the influence of these factors may provide insights into population dynamics of albatross populations and provide a basis for assessing the implications of active management at colonies, such as the eradication of invasive vertebrates.

## Results

### Temporal trends in reproductive rates

**Figure 2:**
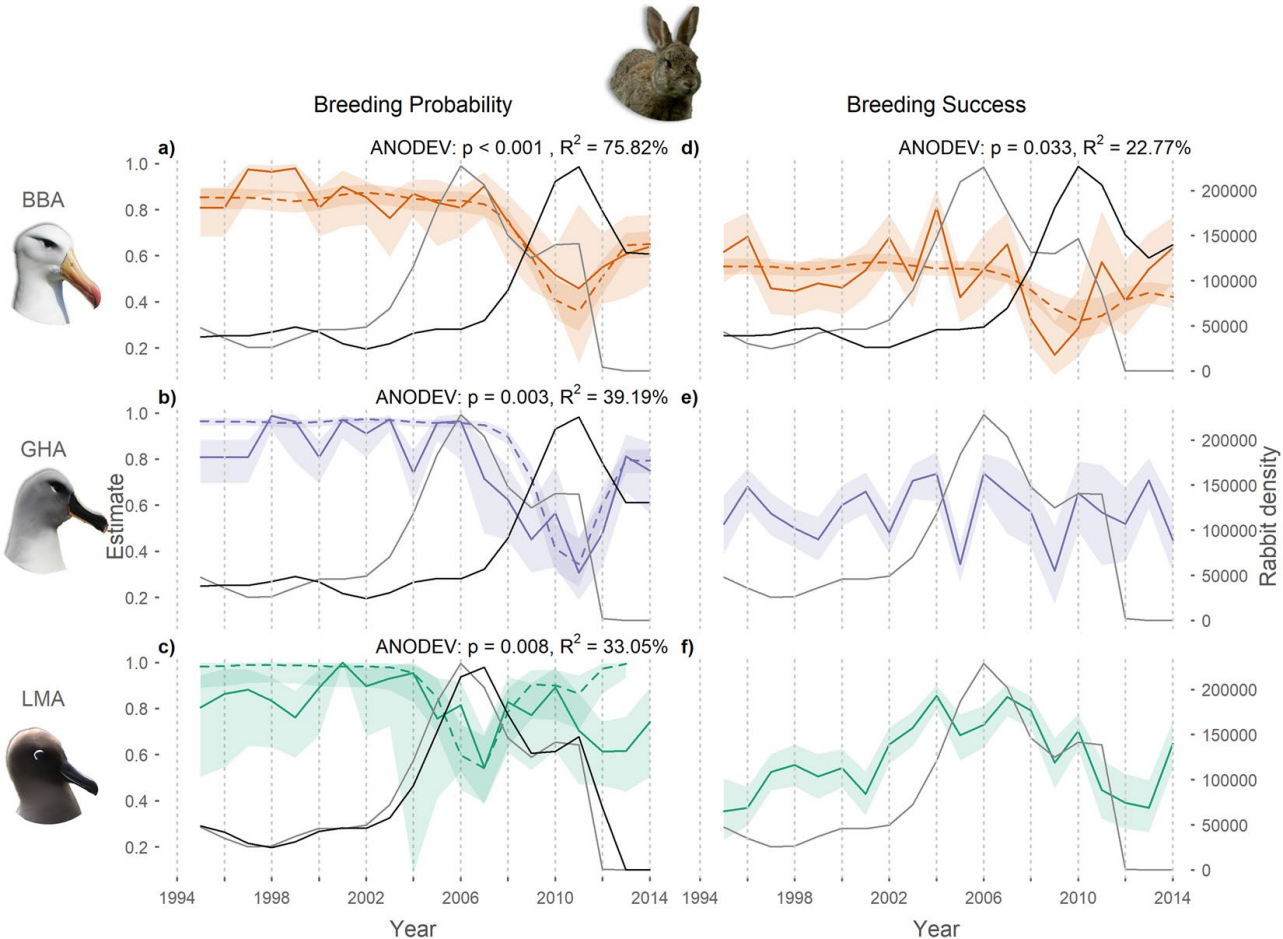
Trends in albatross reproductive parameters and rabbit density. Annual variation in adult breeding probability (**a–c**) and breeding success (**e,f**) of albatrosses at Macquarie Island (black-browed, BBA; grey-headed, GHA; and light-mantled albatrosses, LMA), modelled as time-dependent rates (solid coloured lines) and as a function of the covariate (including 0.95 confidence intervals), lagged island-wide rabbit density (broken coloured lines), overlaid with raw island-wide rabbit density^12^ (raw data, grey solid lines and lagged, black solid lines).

Breeding probability (β) of both black-browed and grey-headed albatrosses decreased over the study period (1995–2014) (Fig. 2a,b and Supplementary Table S1). Although there was a considerable reduction in mean breeding probability between 1995–2004 (mean ± SE: 0.88 ± 0.07) and 2005–2014 (0.73 ± 0.11) of light-mantled albatrosses, a significant trend was not detected, presumably due to high inter-annual variability (Fig. 2c). No trends were detected in breeding success of any species across the study period (see Supplementary Table S1).

### Effects of high rabbit density on reproductive parameters

High island-wide rabbit density was associated with decreases in breeding probability of all three species, explaining 75.8%, 39.2% and 33.1% of the variance for black-browed, grey-headed and light-mantled albatrosses, respectively (Fig. 2a–c). Light-mantled albatrosses, which breed island-wide, showed decreases six months after high rabbit density, whereas black-browed and grey-headed albatrosses, which breed at the southern end of the island, showed a four and a half-year lag between high rabbit density and low breeding probability. When rabbit density (lagged) was highest, the modelled breeding probability of black-browed, grey-headed and light-mantled albatrosses decreased by 35.1%, 50.1% and 26.2%, respectively from the beginning of the study. Following the peak in rabbit density, the subsequent decrease associated with the pest eradication corresponds with a restoration in breeding probability by 18.1%, 44.2% and 19.8% for black-browed, grey-headed and light-mantled albatrosses, respectively.

A negative correlation between rabbit density and breeding success was detected for black-browed albatrosses, explaining 22.8% of the variance of modelled breeding success, and showing the lowest estimate (0.17 ± 0.06) 3 years after peak rabbit density (Fig. 2d). This corresponded with a decrease of 44.7% in modelled breeding success from the beginning of the study. In parallel with the lagged decrease in rabbit density (from 2010 to 2014) was a concurrent increase in black-browed albatross breeding success by 34.8%. There was no effect of rabbit density on breeding success of grey-headed and light-mantled albatrosses (Fig. 2e,f).

### Effects of climatic conditions on reproductive output

**Figure 3:**
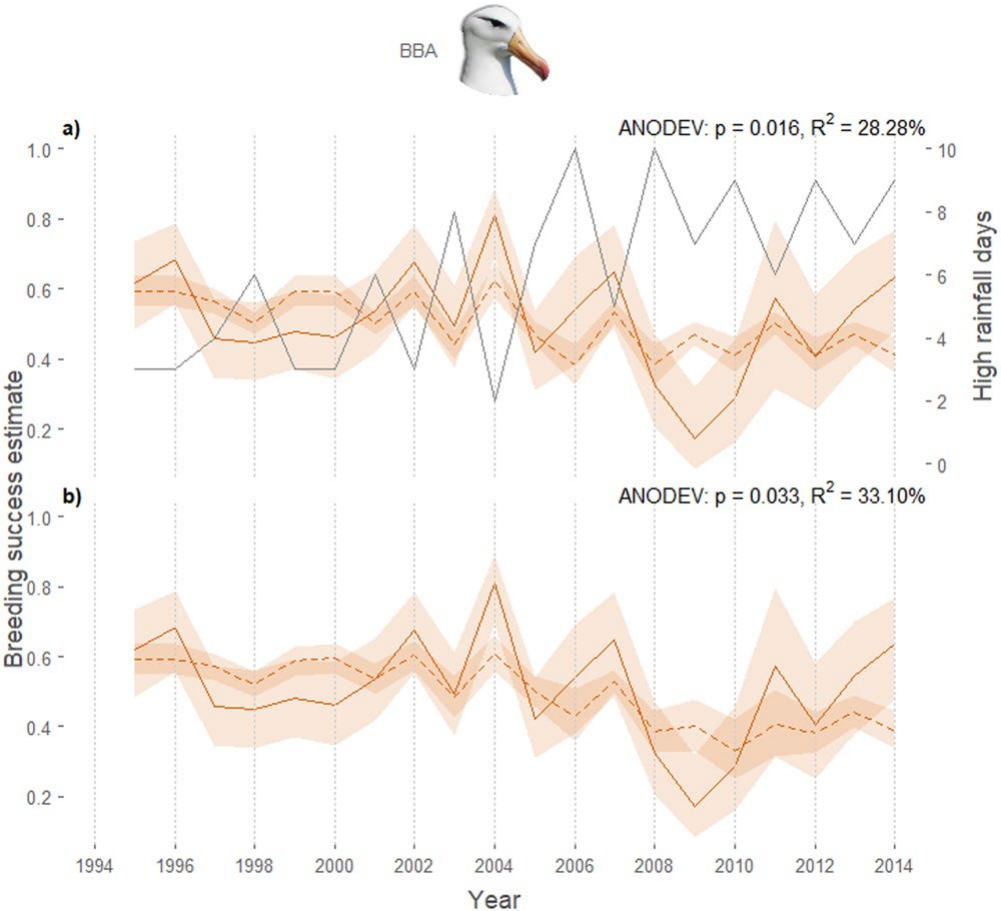
Modelled black-browed albatross breeding success. Annual variation in breeding success probabilities of adult black-browed albatrosses (BBA) at Macquarie Island, modelled as time-dependent breeding success (solid coloured lines) and as a function of the covariates (broken coloured lines, including 0.95 confidence intervals), (**a**) number of January days with total rainfall in the 80^th^ percentile (1995–2014, grey solid lines) and (**b**) combined island habitat model including aforementioned rain covariate and island-wide rabbit density.

The relationship between extreme rainfall on breeding success was only obvious for black-browed albatrosses, explaining 28.3% of the variation (Fig. 3a). The total percentage of temporal variance in breeding success explained by rabbit and rainfall variables combined was 33.1% (Fig. 3b).

### Nest site characteristics

**Figure 4:**
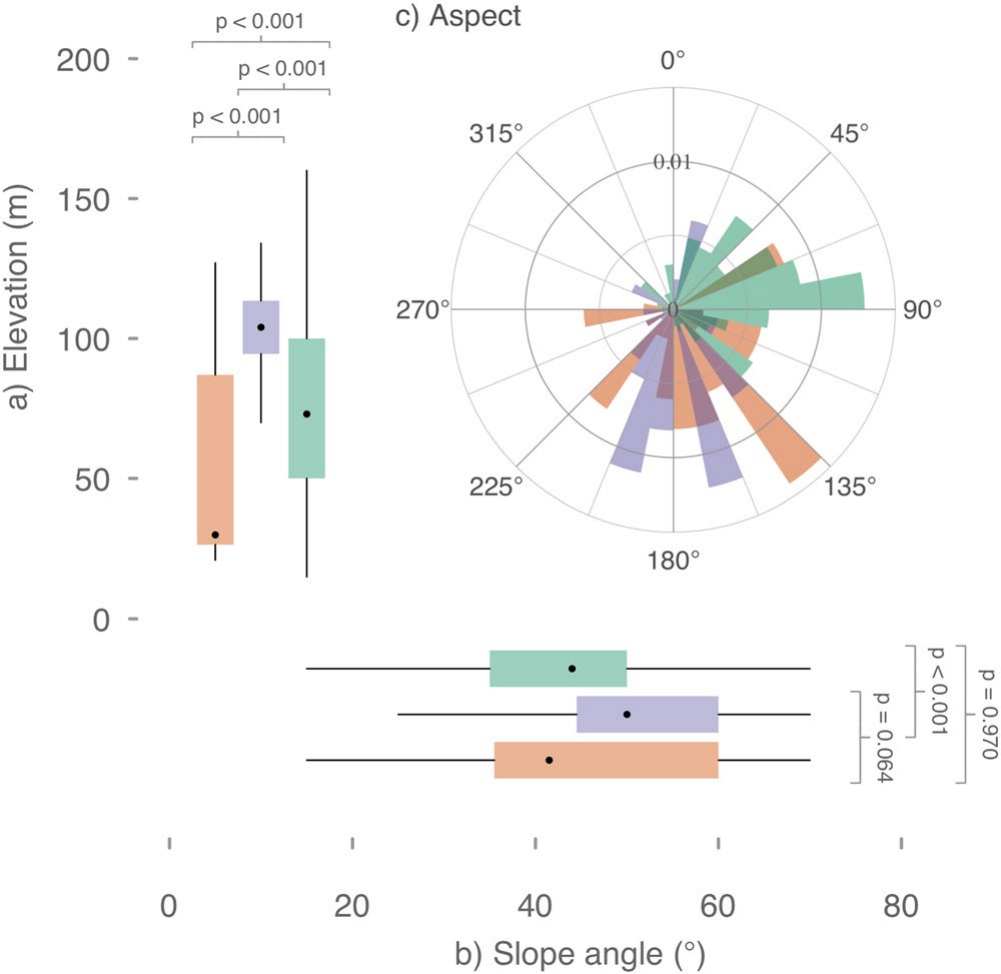
Species-specific nest site characteristics. Observed differences in nest site characteristics of albatrosses at Macquarie Island (black-browed, orange; grey-headed, purple; and light-mantled albatrosses, green) including (**a**) elevation (metres above sea level) (**b**) slope angle and (**c**) nest aspect. The boxplot range reflects 95% CI of the mean and whiskers are 1.5 times the IQR.

There were differences in nest site characteristics among the three albatross species; black-browed albatrosses showed a preference for low elevations (n = 44, $\bar{x}$ = 52.7 ± 5.1 m above sea level) and low slope angles (n = 44, $\bar{x}$ = 44.1 ± 2.4**°**), and grey-headed albatrosses for high elevations (n = 87, $\bar{x}$ = 99.6 ± 2.9 m above sea level) and high slope angles (n = 87, $\bar{x}$ = 49.9 ± 1.4**°**), whereas light-mantled albatrosses showed more variability in elevation (n = 234, $\bar{x}$ = 76.0 ± 2.4 m above sea level) and slope angle (n = 234, $\bar{x}$ = 43.6 ± 0.9**°**) (Fig. 4a,b). These species-specific differences in nest aspect were, in general, related to the topography of Macquarie Island and colony location (F_2,362_ = 140.6, p < 0.001) (Fig. 4c). Colonies of black-browed and grey-headed albatrosses had south-easterly (n = 44, $\bar{x}$ = 159.3 ± 0.1**°)** and southerly (n = 87, $\bar{x}$ = 176.9 ± 0.1°) nest aspects, respectively, reflecting the typical slope aspect at the southern extent of the island. In contrast, light-mantled albatrosses had a broad, island-wide nesting distribution, with the highest concentrations on the east coast where six of seven study sites are located, reflected in the observed nest aspects (n = 234, $\bar{x}$ = 70.3 ± 0.1°).

### Model parameters

Poor model fit was found for all three species; black-browed (χ^2^_95_ = 154.15, p < 0.001), grey-headed (χ^2^_124_ = 390.28, p < 0.001) and light-mantled albatrosses (χ^2^_219_ = 799.70, p < 0.001). For grey-headed and light-mantled albatrosses trap-shyness was detected (χ^2^_21_ = 221.47, z = 10.22, p < 0.001 and χ^2^_29_ = 381.08, *z* = 15.31, p < 0.001, respectively), i.e., where individuals encountered on the first occasion tend to be encountered less at the second occasion. The biennial breeding frequency and associated lower breeding probability of grey-headed and light-mantled albatrosses may explain the trap-shyness detected for these species. Biennial breeding is accounted for in the mark-recapture modelling by possible transitions into unobservable states. In contrast, trap-happiness was detected for black-browed albatrosses (χ^2^_23_ = 88.29, *z* = −6.30, p < 0.001). Trap-happiness, in this case, appears to be an artefact of reduced accessibility for investigators to portions of the study site during periods of high rabbit grazing; consequently, individuals in accessible locations were reencountered more frequently. Black-browed albatross encounter histories showed evidence of underdispersion. The biological explanation for underdispersion is unclear and may simply reflect greater uncertainty in the estimation process due to a smaller sample size. To correct for lack of fit to the underlying capture-mark-recapture assumptions, over dispersion factors (ĉ) of 1.00, 1.00 and 1.46 were included in the models for black-browed, grey-headed and light-mantled albatrosses, respectively^36^.

## Discussion

With a rare insight into the long-term population fluctuations of an invasive species prior to successful eradication we described for the first time the relationship between rabbit grazing and the reproductive output of a threatened albatross community.

At the community level, rabbit density explained a significant proportion of the temporal variation in the reproductive output of the albatross community with reproductive output lowest when the impact of high rabbit density on vegetation and habitat quality was the greatest. Rarely have such colony-based drivers of seabird breeding probability been quantified or shown to explain trait variation for a community of species. Our findings offer support for management intervention, in this case the large-scale eradication of rabbits, which appears to have provided conservation benefits, including a potential increase in reproductive output for an endangered community of albatrosses.

We suggest that the cascading effects of high rabbit density on albatross reproductive output occurred via two, sequential, mechanisms: i) vegetation degradation directly affecting nest site habitat and microclimate over short temporal scales, and ii) vegetation degradation and rabbit burrowing activity reducing soil stability and amplifying natural erosion rates, leading to large-scale degradation of overall slope integrity reduced habitat suitability at breeding colonies.

The greater affect of habitat degradation on breeding probability rather than breeding success is unexpected given that albatrosses are found breeding in exposed colonies with very little vegetation cover (e.g. Steeple Jason Island black-browed albatross colony and The Mewstone shy albatross *Thalassarche cauta* colony). Regardless, albatrosses on Macquarie Island bred in areas of dense tussock grass (*Poa foliosa* and *P. cookii*) during periods of effective rabbit control, until management intervention ceased and heavy rabbit grazing transformed the landscape. Founding nest site selection is likely to be strongly influenced by habitat quality, which for Macquarie Island albatross was drastically and rapidly reduced to dead tussock pedestals, bare ground and mud following increased rabbit numbers. Habitats that provide protection from adverse weather conditions, reduced variability in nest microclimate and cover from predators would, in theory, be selected preferentially^37^. As adults return to colony, and encounter poor nesting conditions, they may either invest in breeding in substandard conditions or skip breeding, consequently maintaining body condition until nest site conditions improve. For Macquarie Island albatrosses, reduced breeding probability during periods of severe rabbit damage potentially reflects their adaptive capacity, whereby the energetic costs of breeding are avoided when the likelihood of reproductive success is low. As long-lived species, albatrosses have many opportunities to breed and can skip some breeding opportunities with little consequence for individual fitness and lifetime fecundity^38^. However, continuous skipping and low breeding probability can have delayed population level consequences by reducing breeding population recruitment rates.

Lower breeding probability may also be influenced by the functional role of vegetation as nesting material. From vegetation photo monitoring of the black-browed and grey-headed albatrosses slopes on Macquarie Island a rabbit driven transition from dominant tall tussock (*P. foliosa* and *P. cookii*) and mega-herb species (*Stilbocarpa polaris*) to bare ground and secondary succession of *Leptinella plumosa* has led to an overall reduction in the availability and quality of nesting material (see Supplementary Figs. S1 and S2). Lack of material potentially increases the energetic cost of nest building, reduces the quality of nests and, in extreme cases, may disrupt courting behaviour, formation of new partnerships, or the likelihood of laying.

The negative relationship between rabbit density and breeding success of black-browed albatrosses may reflect an increase in the metabolic cost of incubation and brood for adults due to reduced vegetative cover and the increased exposure to wind and rain preventing efficient thermoregulation by chicks^39–41^. Furthermore, the loss of nesting habitat through grazing introduces greater variability in microclimate around the nest, and both adult and chick are then more exposed (Fig. 1). A decline in nest quality and slumping associated with reduced availability of nesting material may also explain reduced breeding success. At the Chatham Islands, storm-driven reductions in soil and vegetation from several breeding sites of Chatham (*Thalassarche eremita*) and Northern Royal (*Diomedea sanfordi*) albatrosses has resulted in a considerable decline in hatching success^42^. Furthermore, the loss of vegetation cover might also have led to increased predation on chicks from brown skuas (*Catharacta antarcticus*) and giant petrels (*Macronectes* sp.)^25^. Rabbit densities, vegetation suppression and predation risk impact chick survival through a complex interplay of processes. The eradication of rabbits and subsequent drop in grazing pressure has allowed the vegetation to recover, but also potentially increased predation risk, as skuas lose rabbits as a principal prey resource^25^. The significant correlation between rabbit density and breeding success in black-browed, but not in grey-headed or light-mantled albatrosses, may also be related to the annual breeding strategy of black-browed albatrosses. As annual breeders, black-browed albatrosses face a greater number of years of poor slope condition over the study period. Alternatively, black-browed nests on Macquarie Island predominantly face south-east into the prevailing winds, which may increase the vulnerability of chicks to storm events when vegetation cover is poor.

The asynchronous relationship between breeding probability and rabbit density reflects spatio-temporal heterogeneity in grazing pressure. Prior to this study, the rabbit population on the island was estimated to be several hundred thousand individuals in the mid-1970s, before it was reduced to just tens of thousands around 1997^12^. Following the introduction of myxomatosis in 1978, rabbit numbers plummeted and remained low until the mid-1990s^12,43^. Rabbit numbers increased dramatically from 1998 to 2006^12^ and this boom in rabbit numbers coincided with our study period. This increase reflects growth of localised rabbit populations, particularly in the south of the island, which represents black-browed and grey-headed nesting sites, and at the time showed greater vegetation diversity and abundance^14^. By 2008, heavy grazing had resulted in severe vegetation suppression and slope degradation of the escarpment at both breeding colonies^13^. The four and a half-year lag between high rabbit density on the island and low breeding probability for the two albatross species reflects the delayed vegetation suppression at these southern colonies. For light-mantled albatrosses, which are widely distributed on the island, the half-year lag in the response of breeding probability is more likely to be a direct effect of the high, island-wide rabbit density.

Understanding the effects of invasive species together with that of a changing climate are crucial to mitigating impacts on native species^44^. Subantarctic latitudes have recently experienced changes in predominant weather patterns as a result of global climate change^45–47^. For Macquarie Island, a prolonged positive phase of the Southern Annular Mode has resulted in increases in the frequency and magnitude of heavy rainfall events and increases in average daily wind speeds^6,33^. Extreme rainfall events in January, *i.e*. during the early stages of albatross chick growth, can result in high mortality, as we have shown by the lower breeding success for black-browed albatrosses. Chicks are more vulnerable at young ages because waterproof feathers have not yet developed and they have less ability to thermoregulate^48^. Consequently, protection from extreme events by vegetation, which provides a more stable microclimate, is likely to enhance chick survival. The interaction of high rabbit grazing pressure in an already perturbed system appears an important driver of the low breeding success observed between 2008 and 2010. For black-browed albatrosses the effect of extreme weather may have had a greater influence on breeding success than in other species, because of the lower variation in nest aspect (predominantly south-easterly), low elevation and sparse vegetation in the colony. Breeding birds and their chicks were therefore more exposed to extreme storm events driven by circumpolar deep low-pressure systems, and at greater risk of nest-site waterlogging by escarpment runoff.

The combination of an anomalously high number of extreme rainfall events and high rabbit grazing also presents the possibility of secondary effects on albatross reproductive output, with greater soil instability increasing the frequency of landslips^13,49^. Additionally, increasing wind speeds may accelerate drying of soils between heavy rainfall events, leading to further slope erosion and potentially influence land slipping. However, the stochastic nature and spatial distribution of these events confound efforts to link them statistically to demographic trends.

In this study, the likelihood of incorrectly assigning a breeder as a nonbreeder, or assigning a breeder as not detected is marginally increased during the periods of poor slope condition as the number of accessible nests decreases. To negate the effects of incorrect state assignment detection probability was incorporated in the modelling. Furthermore, the results of the modelling generally show a more significant relationship between rabbit density and albatross breeding probability compared to breeding success. This may in part be explained by the inherent natural variability of each demographic parameter. Breeding probability is generally less variable than breeding success, and in this case only shows a response to rabbit density over the period that corresponds with the greatest rabbit damage. Therefore, the differences in findings may reflect our capacity to detect a relationship for each demographic parameter. The disadvantage of using a correlative approach to estimating drivers of demographic rates is that spurious relationships may be inferred without clear mechanisms of underlying causation. Here we have shown a statistically significant correlation between rabbit densities and reduced breeding probability and success, supported by quantified evidence of high rabbit numbers^12^ and severe habitat degradation^13^.

The effects of non-predatory invasive species on the demography of native species are complex and difficult to quantify. Current research on demographic responses of seabirds to invasive species primarily addresses predation, particularly by cats, rats or mice^50,51^. Furthermore, research on the underlying processes that determine breeding probability of seabirds largely focuses on factors such as breeding experience, adult quality and at-sea conditions^52,53^, with little work on the effects of nesting habitat change.

Prolonged low breeding probability and success, as found on Macquarie Island, is likely to have ramifications for juvenile recruitment in the future, leading to a temporary decrease in the breeding population and a skewed population age structure. Due to a delayed onset of maturity and high recruitment age of albatrosses, the greatest population-level impacts on Macquarie Island may not be observed for 8–14 years after the vegetation recovers.

The success of the Macquarie Island Pest Eradication Project in removing rabbits from the island has resulted in rapid vegetation recovery^32,54^. There are early signs of increases in albatross breeding probability and breeding success, and the continued recovery of slope vegetation should provide greater protection and stability in nest microclimate. However, for black-browed albatrosses, where a large proportion of the population (36.7 ± 1.2%) nest in an area where the dominant tussock species, *P. cookii* exhibits slower growth compared to the widespread *P. foliosa*, recovery of vegetation may take longer. Alternatively, cessation of heavy grazing provides an opportunity for the secondary succession of species with more vigorous recruitment, such as *P. foliosa* or *S. polaris*.

Here we show that breeding site conditions are linked to breeding probability and success. This is important in a conservation context as for wide-ranging species, like albatrosses, apparently indirect onshore management interventions such as removing invasive species, can have a positive effect on vital rates. Affecting change in their at-sea habitats is much more challenging and achieving a conservation outcome correspondingly more difficult and complex^55^. Positive changes in vital rates, as we show, can translate into real outcomes at the population level, such that when invasive species are removed, populations can increase given more individuals are recruited into the potential breeding population by increases in chick fledging rates. By quantifying the indirect and complex effects that invasive species have on the demographics of a community of native seabird species and their subsequent population structure, we obtain greater insight into vulnerability or resilience of each species to changes in ecosystem state and ultimately reduce the likelihood of negative and unintended consequences of future applied conservation management.

## Conclusion

We found that high rabbit density was linked to low breeding probability of subantarctic albatrosses through severe habitat degradation. For one species (black-browed albatrosses), the additive impacts of extreme weather events and high rabbit density explained decreases in breeding success. The future for the albatross populations following the rabbit eradication on Macquarie Island looks positive, given that reproductive ability has already improved. Our findings illustrate the importance of breeding site characteristics on vital rates in albatrosses and show that integrating terrestrial threatening processes is important when assessing population viability and the development of management policy for predominantly marine species.

## Methods

### Study area and field methods

We used 20 years (1995–2014) of capture-mark-recapture data in a multi-event modelling approach^56^ from three albatross species breeding on Macquarie Island (54.6°S, 158.9°E) to quantify the relationship between invasive rabbits and weather patterns, and albatross breeding probability and breeding success.

Macquarie Island is a narrow, elongated, uplifted island oriented approximately north-south. The coastal perimeter of the island rises steeply from sea level to approximately 300 m and provides habitat for escarpment-nesting albatrosses. Albatross construct their nest using nearby material, such as mud, vegetation and other natural debris shaped into a pillar with a rounded hollow on the top that forms the nesting bowl. Adults continue to maintain the nest throughout incubation by adding more organic material.

Between 1995 and 2014 (seasons 1994/95 and 2013/14), all accessible albatross chicks at one black-browed, one grey-headed (encompassing the entire island breeding populations) and seven light-mantled albatross study sites (~10% of total island breeding population) were banded with stainless steel bands. The majority of breeding adults had existing bands from previous long-term studies. Over the study period, annual nest visits during incubation allowed the bands of breeding adults and the presence of an egg to be recorded. Banded nonbreeding adults observed within the study site were also recorded. Nest checks at the end of the breeding season determined whether the chick fledged successfully as a measure of breeding success. All methods were performed in accordance with the relevant guidelines and regulations. Nest monitoring protocols approved by the Tasmanian Department of Primary Industries, Parks, Water and Environment’s Animal Ethics Committee in accordance with the standard operating procedures for seabird research on Macquarie Island.

A geographic survey was undertaken in the 2013/14 season to establish physical differences in elevation, slope angle and aspect of nests of the three species (black-browed, n = 44, grey-headed, n = 87, and light-mantled albatrosses, n = 234). Elevation was measured using a calibrated Garmin GPSMAP 64 s with a built-in barometric altimeter. A Suunto PM-5/360 PC Clinometer was used to determine the slope angle of the escarpment at the nest site. A bearing measurement taken approximately 90° perpendicular to the slope at the nest site using a handheld compass was used to determine nest aspect. Differences in nest site characteristics (aspect, elevation and slope angle) among species were tested using one-way ANOVA and Tukey’s HSD test.

### Reproductive output analysis

An encounter history was produced for each individual (black-browed, n = 225; grey-headed, n = 513; and light-mantled albatrosses, n = 1215) which included several observable adult states, successful breeder (coded 1), failed breeder (coded 2), nonbreeder (coded 3); and unobservable states (coded 0) corresponding to post-successful breeder, post-failed breeder and post nonbreeder (see Supplementary Appendix S1). Unobservable states were included in the analyses to account for the biennial breeding behaviour of grey-headed and light-mantled albatrosses, which results in year-to-year differences in the transitional breeding probability (ψ); the probability of an individual breeding the year following a successful breeding attempt is low, higher after a failed attempt and greatest after a nonbreeding season (see Supplementary Fig. S3)^36^. All chick, juvenile and nonbreeding states before the first breeding attempt were suppressed to focus on reproductive parameters.

Due to the timing of the field seasons, some nests were not revisited late in the season, and this uncertainty in breeding outcome (coded 4) was incorporated in an event matrix^57^, representing 1.5%, 1.1% and 1.3% of observations for black-browed, grey-headed and light-mantled albatrosses, respectively.

To test for goodness-of-fit, encounter histories were transformed to single-state and assessed using U-Care 2.3.2 software, including tests for transience^58,59^. Tests for trap-dependence (Test 2: 2.CT + 2.Cl) were excluded since the model structure accounted for differences in breeding probability based on previous breeding states, characteristic of skip-breeding^36^. A correction for lack of fit within the populations was applied using an over-dispersion factor (ĉ)^60^.

Over the study period, rabbit-driven changes in breeding habitat condition influenced nest accessibility for investigators. Changes in detection probability (*P*) were therefore quantified and incorporated in the demographic models (see Fig. S4). The influence of rabbits on detection was addressed by including rabbit density as a covariate on modelled detection probability (see Supplementary Table S2). Detection probabilities were estimated separately for breeders and nonbreeders, as bands are harder to read on non-nesting birds.

Adult survival (Φ), return rates (*r*), breeding probability (β), breeding success (*γ*), and detection probability (*P*) were estimated using E-Surge 1.9.0 software^61^. See online Supplementary Fig. S3 for probability estimation schematic, Supplementary Appendix S1 for transition matrix structure and Supplementary Table S2 for input notation. For black-browed albatrosses, the breeding probability (β) and breeding success (*γ*) estimates were derived using a time-dependent (*t*) model for all adult observable states due to their annual breeding behaviour. Given the difference in likelihood of carry-over effects on the current state from the previous state of biennial breeders, the probability of return and breeding for successful breeders was different to failed and nonbreeders for the original time-dependent models, except when modelling the demographic parameter of interest, where time-dependent parameters were retained. This constituted our general model for grey-headed and light-mantled albatrosses. Furthermore, because long-term temporal trends in the covariate and demographic rate can obscure their relationship, detrended models that provide a measure of short-term variation were also implemented, which described the total variation explained by the covariates when temporal trends were removed.

Demographic model selection was based on Akaike’s information criterion (AIC), whereby the model with the lowest AIC (ΔQAIC > 2) was chosen as the best model^62^. While it was necessary to address time-dependent variation in adult survival and return probabilities to evaluate reproductive parameters accurately, further interpretation of these is outside the scope of this paper.

### Environmental covariates

Environmental covariates were included in the demographic modelling to quantify the influence of rabbit density and extreme weather events on breeding probability and breeding success. Monthly estimates of island-wide rabbit density (*i.e*. one value for the entire island for each month from 1994 to 2014) were taken from Terauds, *et al*.^12^, who used a smoothed regression model to generate island-wide estimates from rabbit counts in two-hectare study plots. The region-specific rabbit density data represents individual count data shows large inter-annual and intra-annual fluctuations and may obscure any important relationship between albatross breeding. Therefore, modelled island-wide estimates from Terauds, *et al*.^12^ were implemented in the demographic modelling because of the extensive effort this study takes to ensure data accuracy and completeness across the time-series that aligns with the albatross demographic dataset used in this study.

Analyses of breeding probability used averaged monthly rabbit densities during the preceding nonbreeding period, and of breeding success analyses included averaged monthly densities in the corresponding breeding season. Furthermore, it is known that rabbit density was not spatially uniform across the island. High rabbit density regions shifted over the study period resulting in localised patches of severe grazing, and so rabbit impacts were not expected to be synchronous between the three species^14^. Light-mantled albatrosses breed widely across the island whereas breeding colonies of black-browed and grey-headed albatrosses are restricted to the southern end of the island. Peaks in rabbit density at the southern extent of the island, occurred approximately four and a half years later than the peak in island-wide rabbit density and had a more substantial affect on slope stability^12^ (Fig. S5). The impact of high rabbit density is primarily through overgrazing of vegetation, causing direct changes to habitat and subsequent loss of soil stability. These effects occur over variable time scales and so two different lags were used; six months for light-mantled albatross (island-wide colonies) and 4.5 years for black-browed and grey-headed albatrosses (southern colonies) on predictor variables in the models. These lags reflect not only the duration between high rabbit density, vegetation suppression and slope erosion, but also spatio-temporal patterns in grazing.

To investigate the influence of extreme events on breeding success during the early chick-rearing period, when chicks are most vulnerable and thermoregulatory costs are high^48^, heavy rainfall events were included in the modelling. January daily rainfall (mm) values obtained from the Bureau of Meteorology weather station on Macquarie Island were summarised into the number of days above the 80th percentile of the study period average and included as a covariate to capture the frequency of extreme rainfall events. This equated to the number of January days with total rainfall above 4.40 mm.

The significance of each covariate on modelled breeding probability and breeding success was assessed using ANODEV, and the magnitude of its effect determined according to both the total variation explained by the covariates (R^2^) and the total variation explained by the covariates when temporal trends have been removed from the variable and the demographic rate (detrended R^2^) following Grosbois, *et al*.^63^. The detrended models provided a short-term measure of variation compared to the standard models, which consider long-term variability and can be sensitive to temporal trends in the environmental covariate and demographic rate.

## Supplementary information

Supplementary Information.

## Data Availability

The long-term mark-recapture Macquarie Island albatross dataset used in this study is housed by the Marine Conservation Branch within the Department of Primary Industries, Parks Water and Environment, Tasmania, and by the Australian Antarctic Data Centre under the Australian Antarctic program data policy (https://data.aad.gov.au/aadc/about/data_policy.cfm).
