# Supplementary material for: Introduced species and extreme weather as key drivers of reproductive output in three sympatric albatrosses
Source: Sci Rep. 2020 May 18;10:8199. doi: 10.1038/s41598-020-64662-5 (PMC7235215; doi:10.1038/s41598-020-64662-5)
Supplement: Supplementary file 1 — Supplementary Information. [file 41598_2020_64662_MOESM1_ESM.pdf]

Introduced species and extreme weather as key drivers of reproductive output in three sympatric albatrosses

Jaimie B. Cleeland[*a,b], Deborah Pardo[c], Ben Raymond[b,d,a], Aleks Terauds[b], Rachael Alderman[e], Clive R. McMahon[a,f], Richard A. Phillips[c], Mary-Anne Lea[a,d], Mark A. Hindell[a,d]

[a]Institute for Marine and Antarctic Studies, University of Tasmania, 20 Castray Esplanade, Battery Point, Tasmania, 7004, Australia

[b]Australian Antarctic Division, 203 Channel Hwy, Kingston, Tasmania, 7050, Australia

[c]British Antarctic Survey, Natural Environment Research Council, High Cross, Madingley Road, Cambridge, CB3 0ET, UK

[d]Antarctic Climate and Ecosystems CRC, Hobart, TAS 7001, Australia

[e]Department of Primary Industries, Parks, Water and Environment, Hobart, 7000, Tasmania

[f]Sydney Institute of Marine Science, 19 Chowder Bay Road, Mosman, New South Wales, 2088, Australia

* Corresponding author: jaimie.cleeland@gmail.com

Table S1 Demographic model results. Multi-event capture-recapture modelling of black-browed, grey-headed and light-mantled albatrosses at Macquarie Island, testing the influence of island-wide rabbit density on breeding probability, and rabbit density, extreme January rainfall events (80th percentile) on breeding success in 1994-2015.

		Model	N_p	qAIC	Deviance	*p* ANODEV	R^2 (%)	Slope (±SE)
**Breeding probability (β)**	*Black-browed albatross*							
		cst	1	6136.2	5958.2			
		t	20	6082.99	5868.99			
		t + lin	2	6073.55	5893.55	**<0.001**	72.47	-0.89±0.15
		t + rabbit$_{lag4.5}$	2	6070.56	5890.56	**<0.001**	75.82	-0.8±0.1
		t + lin + rabbit$_{lag4.5}$	3	6067.24	5885.24	**0.009**	33.82	-0.31±0.1
	Grey-headed albatross							
		cst	1	10681.65	10539.65			
		t	20	10589.95	10409.95			
		t + lin	2	10634.22	10490.22	**0.004**	38.11	-1.17±0
		t + rabbit $_{lag4.5}$	2	10632.82	10488.82	**0.003**	39.19	-1.35±0.11
		t + lin + rabbit $_{lag4.5}$	3	10624.72	10478.72	0.11	14.33	-0.59±0.11
	Light-mantled albatross							
		cst	1	20111.77	29138.34			
		t	20	20111.08	29081.87			
		t + lin	2	20108.04	29129.98	0.094	14.8	-0.95±0.41
		t + rabbit $_{lag0.5}$	2	20100.98	29119.67	**0.008**	33.05	-1.51±0.38
		t + lin + rabbit$_{lag0.5}$	3	20099.35	29114.37	**0.011**	32.45	-1.24±0.33
**Breeding success (γ)**	*Black-browed albatross*							
		cst	1	6132.9	5956.9			
		t	20	6082.99	5868.99			
		t + lin	2	6130.86	5952.86	0.364	4.6	-0.14±0.07
		t + rabbit $_{lag4.5}$	2	6114.88	5936.88	**0.033**	22.77	-0.34±0.08
		t + lin + rabbit$_{lag4.5}$	3	6112.3	5932.3	**0.031**	24.51	-0.34±0.08
		t + rain	2	6110.04	5932.04	**0.016**	28.28	-0.32±0.07
		t + lin + rain	3	6108.35	5928.35	**0.017**	29.21	-0.32±0.07
		t + rabbit $_{lag4.5}$ + rain	3	6107.8	5927.8	**0.033**	33.1	
		t + lin + rabbit $_{lag4.5}$ + rain	4	6092.68	5910.68	**0.004**	50.29	
	Grey-headed albatross							
		cst	1	10644.28	10500.28			
		t	20	10587.64	10405.64			
		t + lin	2	10646.2	10500.2	0.901	0.09	0.02±0.06
		t + rabbit$_{lag4.5}$	2	10645.57	10499.57	0.715	0.76	-0.06±0.07
		t + lin + rabbit$_{lag4.5}$	3	10645.79	10497.79	0.514	2.55	-0.1±0.06
		t + rain	2	10645.72	10499.72	0.746	0.6	0.04±0.06
		t +lin + rain	3	10647.65	10499.65	0.756	0.58	0.04±0.05
	Light-mantled albatross							
		cst	1	20119.58	29146.83			
		t	20	20106.29	29071.95			
		t + lin	2	20120.14	29144.72	0.479	2.82	0.06±0
		t + rabbit$_{lag0.5}$	2	20120.82	29145.63	0.594	1.61	0.01±0.1
		t + lin + rabbit$_{lag0.5}$	3	20121.51	29146.72	0.873	0.15	-0.01±0
		t + rain	2	20122.12	29144.69	0.936	0.04	-0.01±0.09
		t + lin + rain	3	20120.18	29141.87	0.416	3.92	-0.06±0.04

* Terms are N_p, the number of structural parameters, ANODEV, analysis of deviance (Grosbois et al., 2008), R^2, the proportion of variance explained by the test covariate. The three standardised environmental covariates used are *rabbit*, mean monthly modelled island-wide rabbit density (Terauds et al., 2014), *rain*, number of January days of high rainfall (80th percentile of 1995-2014 data) and *wind*, number of January days with high wind events (80th percentile of 1995-2014 data). Models *cst*, *t*, *t + lin* and *t + lin +* covariate, correspond to constant, time-dependent, linear and detrended models tested on reproductive rates of breeding probability (β) and breeding success (γ).

Table S2 Input GEMACO sentences for the time-dependent model analysis in E-Surge. ‘t’ refers to time and ‘f’ refers to previous breeding state, 1 to 63 refers to time periods 1952 to 2015 + others accounts for the parameters not accounted for in the main sentence, ‘i’ stands for constant (refer to E-Surge manual). The ‘firste’ and ‘nexte’ notation refers to the first and next events. Seasons 1994/95 to 2014/15 correspond time steps 43 to 63 and are the subject of the analysis.

		Black-browed albatross	Grey-headed albatross	Light-mantled albatross
**Initial state (*i*)**		i	i	i
**Transition (ψ)**	Survival (ϕ)	t(1:42) + t(43_63)	t(1:42) + t(43_63)	t(1:42) + t(43_63)
	Return (r)	t(1:42) + t(43_63)	[t(43_63) + f(1,2 3 4 5 6)] + others	[t(43_63) + f(1,2 3 4 5 6)] + others
	Breeding (β)	t(1:42) + t(43_63)	[t(43_63) + f(1,3 5 7 8 9)] + others	[t(43_63) + f(1,3 5 7 8 9)] + others
	Breeding success (γ)	t(1:42) + t(43_63)	t(1:42) + t(43_63)	t(1:42) + t(43_63)
**Event (Ω)**	Detection (P)	firste + nexte.[t(43_63) + f(1 2,3)] + others	firste + nexte.[t(1:42) + f(1 2,3).[i + t(43_62) * rabbit $_{lag4.5}$]]	firste + nexte.[f.[t(1:42) + [i + t(43_62) * rabbit $_{lag0.5}$]]]
	Breeding outcome uncertainty (k)	i	i	i

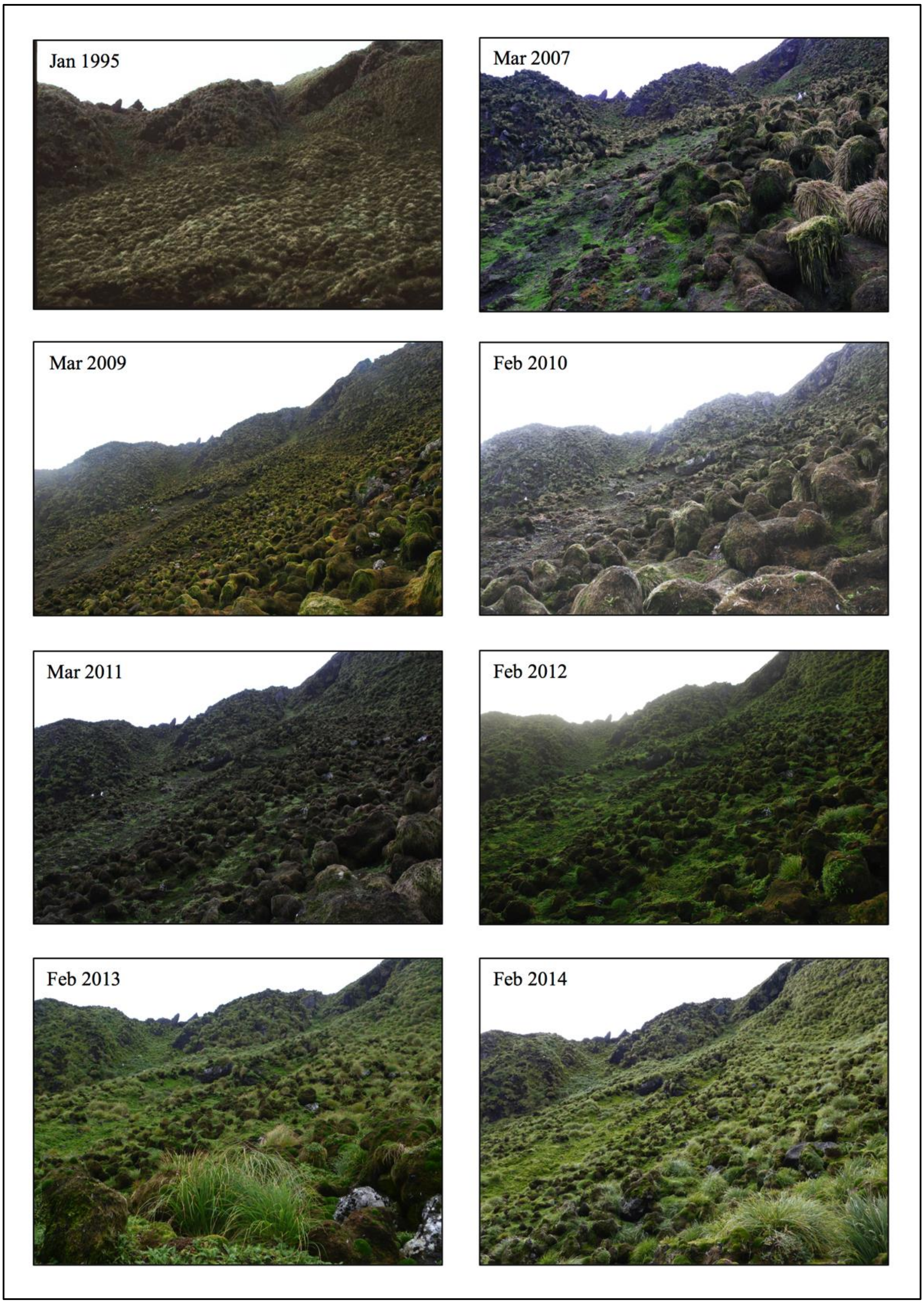

Figure S1 Temporal habitat change in albatross breeding areas on Macquarie Island. Time series (1995-2014) of rabbit driven degradation at the black-browed and grey-headed breeding colony the south-west slopes of Macquarie Island. The images taken from March 2007 show a large scar from a landslide triggered by heavy vegetation suppression caused by rabbit grazing. Images sourced from J. Scott.

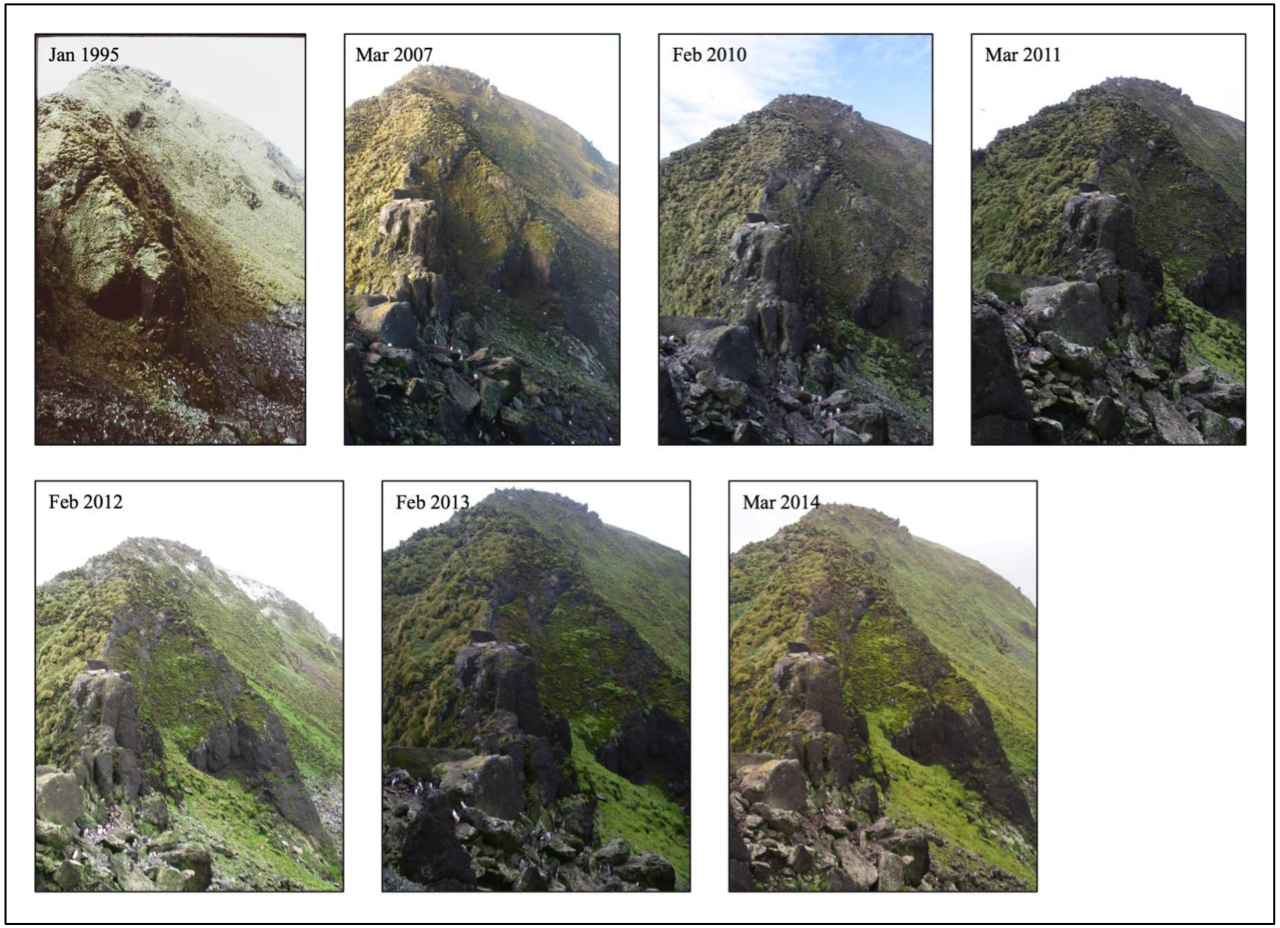

Figure S2 Temporal habitat change in albatross breeding areas on Macquarie Island. Time series (1995-2014) of rabbit driven degradation at the black-browed and grey-headed breeding colony the south-west slopes of Macquarie Island. The bottom right quadrant of each image represents the main breeding area of black-browed albatross. The first image taken in January 1995 shows tussock *Poa foliosa* as the dominant vegetation in this area, by March 2007 heavy rabbit grazing saw a transition to dead tussock and mud which is followed by the slow colonization of this area by *Leptinella plumosa* (2010-2014). Images sourced from J. Scott.

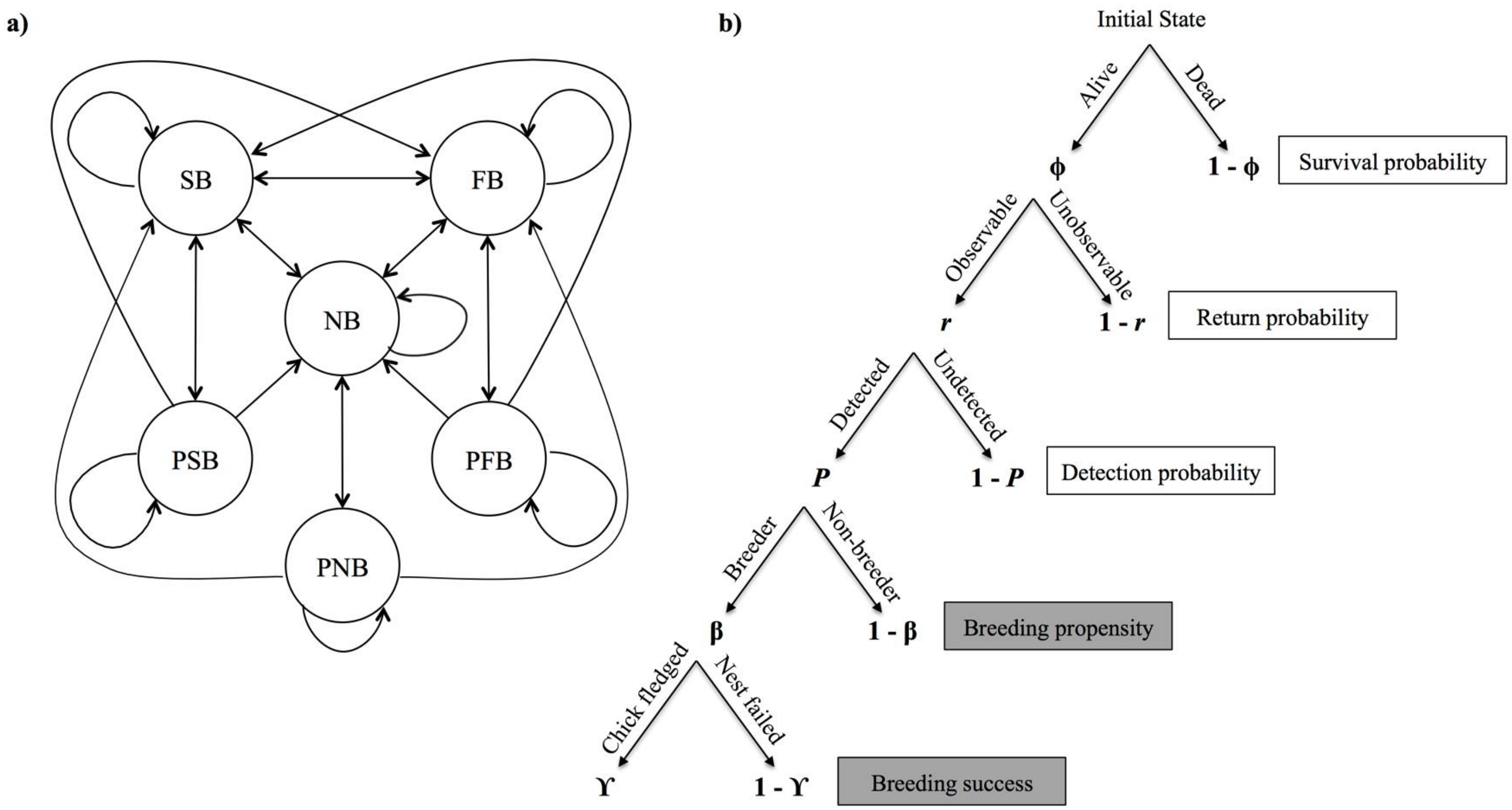

Figure S3. Demographic model structure a) Possible state transition pathways of adult albatrosses at Macquarie Island (black-browed, grey-headed and light-mantled albatrosses) including observable states; successful breeder (SB), failed breeder (FB), nonbreeder (NB); and unobservable states, post-successful breeder (PNB), post-failed breeder (PFB) and post nonbreeder (PNB). b) Probability estimation of demographic parameters of individual survival (ϕ), return (r), detection (P), breeding (β) and success (γ) associated with progressing from the initial state (encountered as SB or FB) to fledging a chick.

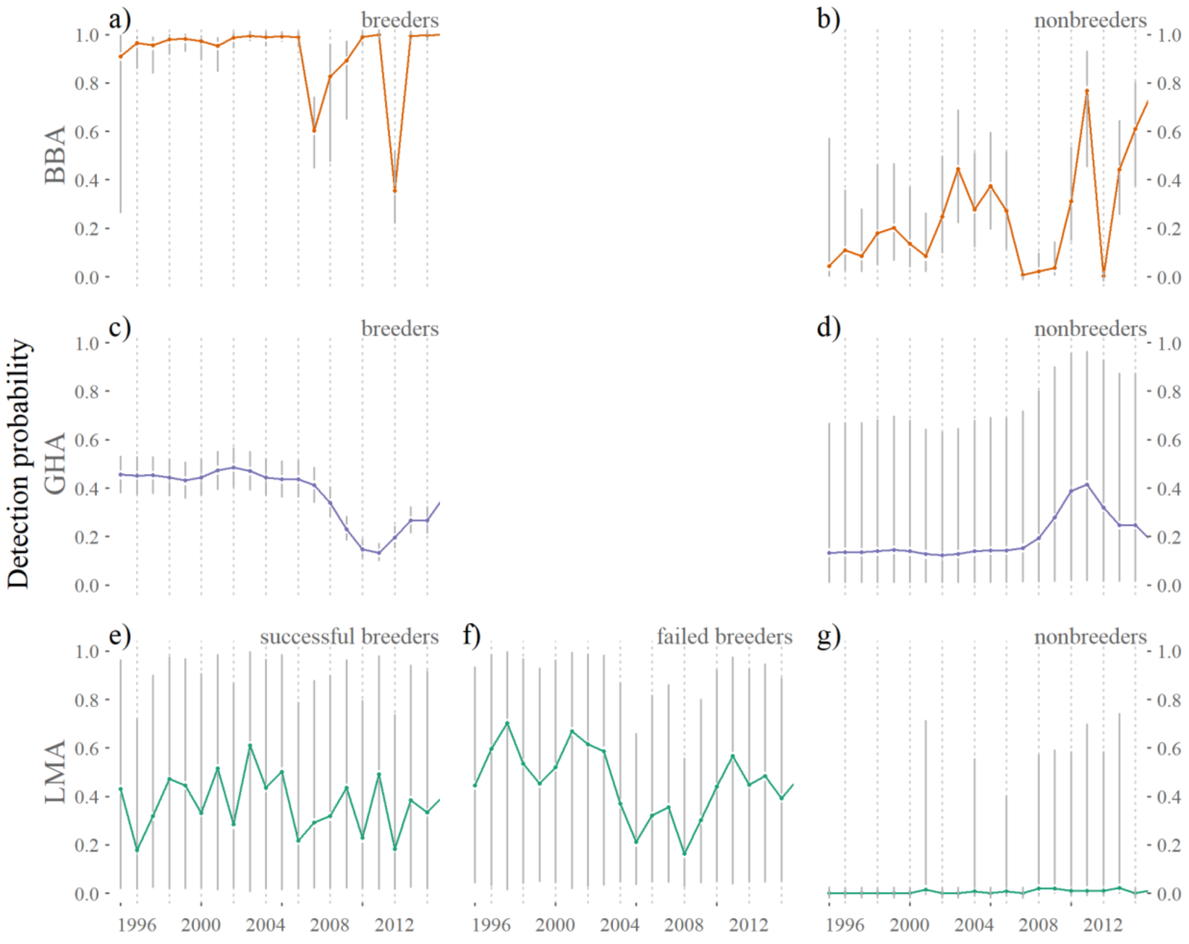

Figure S4 Detection probability related to experimental procedures. Annual variation in detection probability of experienced adult breeders at Macquarie Island (black-browed, BBA, orange; grey-headed, GHA, purple; and light-mantled albatrosses, LMA, green), modelled as time-dependent (solid coloured lines) and grouped by breeding status (including failed breeders for light-mantled albatrosses).

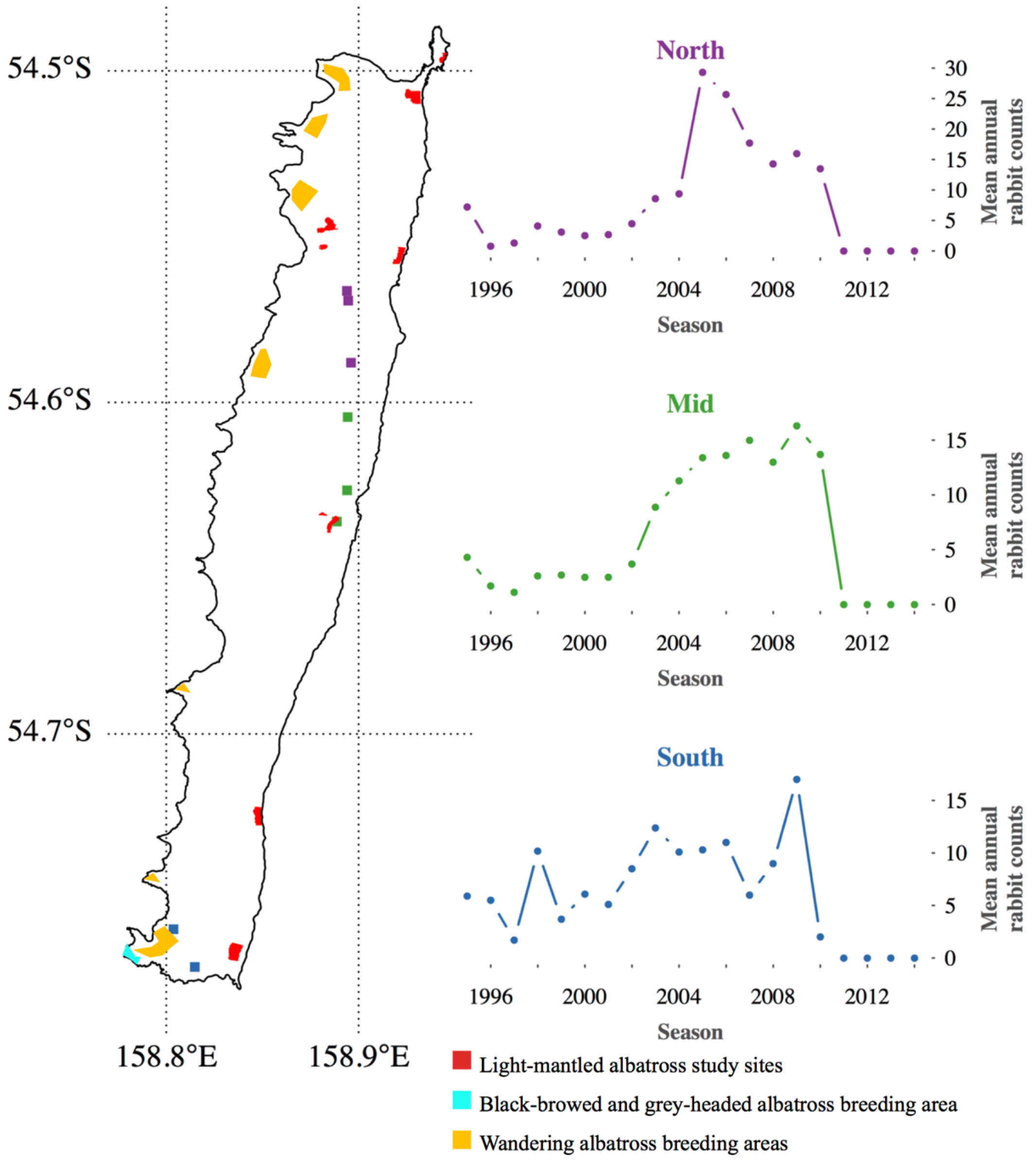

Figure S5 Spatiotemporal patterns in rabbit density. Data from the northern (purple squares), mid (green squares) and southern (blue squares) rabbit count areas (annual mean of monthly rabbit counts from 1 ha plots) in relation to albatross breeding areas on subantarctic Macquarie Island (Terauds et al. 2014).

Appendix S1 Input GEPAT matrices for analysis in E-Surge.

Initial State (i)

$$i = [i \quad 1-i \quad - \quad - \quad - \quad -]$$

Survival (ϕ) transition:

$$\phi_t\psi = \begin{bmatrix} \phi & - & - & - & - & - & 1-\phi \\ - & \phi & - & - & - & - & 1-\phi \\ - & - & \phi & - & - & - & 1-\phi \\ - & - & - & \phi & - & - & 1-\phi \\ - & - & - & - & \phi & - & 1-\phi \\ - & - & - & - & - & \phi & 1-\phi \\ - & - & - & - & - & - & \dagger \end{bmatrix}$$

Return (r) transition:

$$r_t\psi = \begin{bmatrix} r & 1-r & - & - & - & - & - & - & - & - \\ - & - & r & 1-r & - & - & - & - & - & - \\ - & - & - & - & r & 1-r & - & - & - & - \\ - & 1-r & - & - & - & - & r & - & - & - \\ - & - & - & 1-r & - & - & - & r & - & - \\ - & - & - & - & - & 1-r & - & - & r & - \\ - & - & - & - & - & - & - & - & - & \dagger \end{bmatrix}$$

Breeding (β) transition:

$$\beta_t\psi = \begin{bmatrix} \beta & 1-\beta & - & - & - & - & - & - & - & - & - & - & - \\ - & - & 1 & - & - & - & - & - & - & - & - & - & - \\ - & - & - & \beta & 1-\beta & - & - & - & - & - & - & - & - \\ - & - & - & - & - & 1 & - & - & - & - & - & - & - \\ - & - & - & - & - & - & \beta & 1-\beta & - & - & - & - & - \\ - & - & - & - & - & - & - & - & 1 & - & - & - & - \\ - & - & 1-\beta & - & - & - & - & - & - & \beta & - & - & - \\ - & - & - & - & - & 1-\beta & - & - & - & - & \beta & - & - \\ - & - & - & - & - & - & - & - & 1-\beta & - & - & \beta & - \\ - & - & - & - & - & - & - & - & - & - & - & - & \dagger \end{bmatrix}$$

Breeding success (γ) transition:

$$\gamma_t\psi = \begin{bmatrix} \gamma & 1-\gamma & - & - & - & - & - \\ - & - & 1 & - & - & - & - \\ - & - & - & 1 & - & - & - \\ \gamma & 1-\gamma & - & - & - & - & - \\ - & - & 1 & - & - & - & - \\ - & - & - & - & 1 & - & - \\ \gamma & 1-\gamma & - & - & - & - & - \\ - & - & 1 & - & - & - & - \\ - & - & - & - & - & 1 & - \\ \gamma & 1-\gamma & - & - & - & - & - \\ \gamma & 1-\gamma & - & - & - & - & - \\ \gamma & 1-\gamma & - & - & - & - & - \\ - & - & - & - & - & - & \dagger \end{bmatrix}$$

Detection (P) event:

$$P_t\Omega = \begin{bmatrix} 1-P & P & - & - \\ 1-P & - & P & - \\ 1-P & - & - & P \\ 1 & - & - & - \\ 1 & - & - & - \\ 1 & - & - & - \end{bmatrix}$$

Breeding outcome uncertainty (k) event:

$$k_t\Omega = \begin{bmatrix} 1 & - & - & - & - \\ - & 1-k & - & - & k \\ - & - & 1-k & - & k \\ - & - & - & 1 & - \end{bmatrix}$$
